# A Novel Dietary Intervention Reduces Circulatory Branched-Chain Amino Acids by 50%: A Pilot Study of Relevance for Obesity and Diabetes

**DOI:** 10.3390/nu13010095

**Published:** 2020-12-30

**Authors:** Imran Ramzan, Moira Taylor, Beth Phillips, Daniel Wilkinson, Kenneth Smith, Kate Hession, Iskandar Idris, Philip Atherton

**Affiliations:** MRC-Versus Arthritis Centre for Musculoskeletal Ageing Research, National Institute for Health Research Nottingham Biomedical Research Centre, Clinical, Metabolic and Molecular Physiology, University of Nottingham, Royal Derby Hospital, Derby DE22 6DT, UK; imran.ramzan@nottingham.ac.uk (I.R.); moira.taylor@nottingham.ac.uk (M.T.); beth.phillips@nottingham.ac.uk (B.P.); Daniel.Wilkinson@nottingham.ac.uk (D.W.); ken.smith@nottingham.ac.uk (K.S.); kate.hession@ucdconnect.ie (K.H.)

**Keywords:** branched-chain amino acid, essential amino acids, non-essential amino acids, restriction, dietary, maple syrup urine disease, iso-nitrogenous, iso-caloric

## Abstract

Elevated circulating branched-chain amino acids (BCAAs; isoleucine, leucine, and valine) are associated with obesity and type 2 diabetes (T2D). Reducing circulatory BCAAs by dietary restriction was suggested to mitigate these risks in rodent models, but this is a challenging paradigm to deliver in humans. We aimed to design and assess the feasibility of a diet aimed at reducing circulating BCAA concentrations in humans, while maintaining energy balance and overall energy/protein intake. Twelve healthy individuals were assigned to either a 7-day BCAA-restricted diet or a 7-day control diet. Diets were iso-nitrogenous and iso-caloric, with only BCAA levels differing between the two. The BCAA-restricted diet significantly reduced circulating BCAA concentrations by ~50% i.e., baseline 437 ± 60 to 217 ± 40 µmol/L (*p* < 0.005). Individually, both valine (245 ± 33 to 105 ± 23 µmol/L; *p* < 0.0001), and leucine (130 ± 20 to 75 ± 13 µmol/L; *p* < 0.05), decreased significantly in response to the BCAA-restricted diet. The BCAA-restricted diet marginally lowered Homeostatic Model Assessment of Insulin Resistance (HOMA-IR) levels: baseline 1.5 ± 0.2 to 1.0 ± 0.1; (*p* = 0.096). We successfully lowered circulating BCAAs by 50% while maintaining iso-nitrogenous, iso-caloric dietary intakes, and while meeting the recommended daily allowances (RDA) for protein requirements. The present pilot study represents a novel dietary means by which to reduce BCAA, and as such, provides a blueprint for a potential dietary therapeutic in obesity/diabetes.

## 1. Introduction

An elevated blood concentration of branched-chain amino acids (BCAAs) (isoleucine, leucine, and valine) is an established biomarker and prognostic factor for obesity, insulin resistance, and type 2 diabetes (T2D) [1,2,3]. Indeed, evidence from clinical and pre-clinical studies has long supported the notion that BCAAs are a root cause of insulin resistance via multiple mechanisms [4] involving the liver [5], skeletal muscle [6] and adipose tissue [7]. Exemplifying this, experimental BCAA restriction in Zucker rats by ~45% rescued insulin resistance and glycogen storage [8]. Similarly, reducing BCAA was shown to promote fat loss and increased glucose tolerance in obese mice [9], and in obese (ob/ob) mice, increasing BCAA catabolic flux using an inhibitor of Branched-chain alpha-keto acid dehydrogenase kinase resulted in reduced levels of BCAAs and decreased insulin resistance [10]. Collectively, these data suggest that targeting dysregulation of BCAAs by reducing circulating BCAA levels could offer important therapeutic value.

Nonetheless, the feasibility of conducting a human, controlled BCAA-restriction intervention that is food-based with bespoke iso-nitrogenous and iso-caloric content is a challenge and remains largely untested. Indeed, BCAA dietary restriction studies in humans are limited to only two studies. The first was a 4-day BCAA-restriction study in six healthy volunteers which reported only modest changes in plasma BCAA levels [11] and the second, more recently, a one-week dietary restriction of BCAAs in 12 patients with T2D during a 4-week iso-caloric diet [9]. Although the latter study reported an enhancement in postprandial insulin secretion, alongside an improvement in white adipose tissue metabolism and gut microbiome composition following BCAA restriction, a dietary iso-nitrogenous and iso-caloric state was not achieved during the period of BCAA restriction. This limits interpretation since any reported effects cannot be asserted as solely due to BCAA restriction. Moreover, a failure to maintain an iso-nitrogenous diet may limit its utility and longer-term clinical translational relevance due to potential adverse impact of essential amino acid restriction e.g., upon skeletal muscle [10].

To this end, we designed a novel strategy to restrict BCAAs by adapting the dietary management principles used in Maple Syrup Urine Disease (MSUD) [11]. A diet plan was designed and adjusted proportionally to meet each participant’s energy requirement, which adhered to dietary advice for T2DM (i.e., energy from carbohydrate, fat and protein 50, 35 and 15% respectively), while delivering sufficient total protein but with restricted levels of BCAAs.

## 2. Methods

### 2.1. Study Ethics and Participants

All participants gave written informed consent before inclusion in the study in accordance with the Declaration of Helsinki, and approved by University of Nottingham Ethics committee, reference number 158–1711 approved on the 24th of November 2017. We excluded participants who presented with metabolic, respiratory, or cardiovascular disorders, or who were prescribed medication (e.g., beta-blockers, statins and anti-inflammatory drugs), and participants who were not habitual meat (and/or) fish and dairy consumers. Of the screened participants, 12 healthy, normotensive (<140/90) male and females were assigned into a single center, single-blind one-week nutritional intervention study. All participants were monitored throughout the study by an experienced dietitian registered with the Health & Care Professions Council (HCPC). No adverse events were reported during or after completion of the study. Participants were followed for 4 weeks after study completion.

### 2.2. Study Protocol

All volunteers were asked to attend the clinical investigation room at the Clinical, Metabolic, and Molecular Physiology research laboratories at approximately 0830 h, with not eaten for 12 h. Each volunteer had a total of four visits (screening, day 1, day 3 and day 7) for venepuncture and dietary discussions.

Screening: Volunteers attended for a screening visit where they completed a medical questionnaire, physical examination, routine blood chemistry, resting electrocardiogram, and completed a 4-day diet diary. After successfully completing the screening volunteers were assigned to either the BCAA-restricted diet or the control diet for 7 days.

DAY 1: Fasting BCAA levels were quantified in a blood sample obtained via venepuncture. Height, weight, and blood pressure was also recorded. Volunteers were then provided with either the BCAA-restricted or the control diet to consume for the next 7-days. All volunteers received pre-cooked and pre-weighed frozen food with cooking instructions on how to heat the food.

DAY 3: The food diary of the first 2 days was collected. Weight, blood pressure and fasting BCAA levels were again recorded (again via venepuncture).

DAY 7: The procedures from day 3 were repeated but with the diet diary containing information on the previous 4 days.

### 2.3. BCAA Restriction and Control Diet Plan Construction

Both the BCAA-restricted diet and control diets adhered to principles for those with type 2 diabetes and met individual energy and protein requirements. They were constructed in such a way as to assure results between the study and control group diets were caused solely by differing percentages of BCAAs and were not confounded by other factors. This was achieved by using foods with a low BCAA to total protein ratio, specially modified low protein food products and medical supplements developed for the treatment of MSUD, in the diet of both groups. The BCAA-restricted diet plan and the control diet plan each consist of the same menu of foods (3 meals and 3 snacks per day; repeated on each of the 7 days) (Table 1) but with different liquid, amino acid supplements consumed at the main meals and evening snack.

The estimated energy requirement of each participant was met, to ensure weight maintenance and prevent a catabolic state. Individual energy requirement estimates were based on resting metabolic rate (RMR) × physical activity levels (PAL) [12]. The Short-form International Physical Activity Questionnaire (IPAQ) was used to refine the estimate of PAL. The diet adhered to the recommendations for the treatment of type 2 diabetes, (i.e., percentage of total dietary energy from carbohydrate, fat and protein 50%, 35% and 15% respectively) [13] and met the requirement for 0.75g of protein per kg [13]. The 75% restricted BCAA target of 4.2% of energy from protein was derived from observation of the typical diet of omnivores in the UK, in which approximately 16.9% of total protein energy is from BCAAs [14]. The targets, in the restricted diet, for percentage of total energy from protein for each of the BCAA were as follows: Isoleucine 1.1%; leucine 1.8% and valine 1.3%; again, based on levels seen in a typical omnivorous UK diet (isoleucine 4.3%; leucine 7.5% and valine 5.15% of total protein energy). To maximize the amount of total dietary protein provided by food, while achieving the prescribed restriction in BCAAs, foods with naturally higher proportions of BCAA to total amino acids (e.g., cereals, meat, dairy products) were severely restricted, and foods with a naturally low BCAA to total amino acid ratio (e.g., fruit and vegetables) were prescribed [15]. In addition, it was assumed that approximately 16% of protein from vegetables and fruit is derived from BCAA. The menu was then refined by adjusting the quantity of individual food items to ensure that the target for each of the BCAA acids was achieved.

The liquid amino acid supplements differed between study arms in amino acid composition but NOT total amino acid amount; and with the food component met the 24 h amino acid (protein) requirement. Both the BCAA-restricted liquid supplement and the control liquid supplement were built around the BCAA free, amino acid drink MSUD Lophlex LQ 20 (Nutricia), with added glutamine, asparagine, and glutamate. BCAAs are key precursors for the synthesis of alanine, glutamate, and glutamine, which are key in many metabolic processes including energy production, gluconeogenesis, immune cells, and gut turnover. Given that alanine, glutamate, and glutamine are not included in the BCAA free supplement it was important to include them, through their inclusion we were aiming to avoid further depletion of the BCAA pool. With each 125mls of MSUD Lophlex LQ 20 (which provides 20g of protein) the following quantities of amino acid were provided: Asparagine 0.73 g, Glutamate 1.9 g, Glutamine 1.13 g (Table 1).

The control group supplement also contained sufficient BCAAs to ensure that the total BCAA intake (diet component plus liquid supplement) was equivalent to a non-BCAA-restricted diet in which there would be 16.9% of total protein energy from BCAA. The control group received the same Lophlex supplement as the BCAA-restricted group with the addition of isoleucine 1.3 g, leucine 2.1 g and valine 1.5 g.

The supplements were consumed with each of the three meals in quantities that reflected the distribution of protein across the day (Breakfast 34%, Lunch 21%, Dinner 27% and Evening Snack 19%). (The mid-morning and mid-afternoon snack each contained less than 1% of the dietary protein and were served without a supplement). Participants were provided with pre-weighed amino acids for each Lophlex sachet, they simply emptied the contents into a clear jar mix well and drank the required amount (Table 1).

For the food element of this study naturally very low/no protein foods and commercially produced dietetic very low/ no protein foods (Loprofin bread, Loprofin mix, Loprofin low protein rice or pasta and SnoPro: Nutricia) were then included in the same amounts in each arm to ensure that the food component plus the liquid supplement component met other dietary targets, such as energy, carbohydrate, and fat. This dietary program was overseen by an experienced HCPC registered dietitian.

All the curry, soup, and muffins for the 7 days for each participant were produced in a batch specific to the individual, and divided into six equal daily portions, thus avoiding any error associated with estimating weight change during cooking. It was assumed that the composition of the main dietary elements of interest were stable during cooking. The curry and soup were chosen to facilitate a homogeneous distribution of ingredients, hence minimizing between day differences in amino acid delivery; and were easy, safe, and appropriate for regeneration, using a microwave with minimal loss of palatability (clear storage and reheating instructions were provided to ensure microbial safety). All other items were provided in meal or snack portions and had been selected to be easily and safely consumed outside the home, if necessary (Table 1).

An interactive Excel spreadsheet was used to establish each participant’s weight specific requirements for foods, Lophlex, and added amino acids. Subsequently, duplicate samples of soup, curry and muffins were analyzed for BCAA acid content and other key nutrients to allow comparison with the estimate composition. There was no between-participant or between-intervention arm differences in the types of foods or macronutrient contents except for proportional adjustments to all items made to reflect the energy requirements of the participant).

### 2.4. Amino Acid Quantification

To measure the concentration of all plasma amino acids, including BCAA, we separated red cells from the plasma by centrifugation at 3000× *g* for 20 min at 4 °C with the plasma aliquoted and stored at −20 °C until analysis. To determine AA concentrations, we added stable isotopically labelled internal standards and prepared samples as per our standard method [16]. Briefly, plasma proteins were precipitated with 1 mL ice-cold ethanol and centrifuged at 1000 rpm for 5-min, removed the supernatant and evaporated to dryness at 90 °C using nitrogen, followed by re-suspension in 0.5 M hydrochloric acid (HCl). Ethyl acetate was then added, and samples were vortexed thoroughly before upper, ethyl acetate layer (containing bvlipids) was extracted. The aqueous AA-containing layer was evaporated to dryness under a steady flow of nitrogen at 90 °C. Derivatization of the dry residue achieved via addition of equal volumes of Dimethylformamide (DMF) N-Methyl-N-(tert-butyldimethylsilyl) trifluoroacetamide (MTBSTFA), and incubated at 90 °C for 45 min thus converting the AA’s to their t-BDMS derivatives. AA concentrations were determined with reference to a calibration curve composed of standard amino acid mix of known quantity and analyzed by GC-MS. To quantify plasma AA concentration, 0.5 uL of sample was injected into a Series Single Quadrupole (ISQ) Trace 1300 single quadrupole GC-MS (ThermoFisher Scientific, Hempel Hempstead, UK). A split injection mode (1:10) was used, at an initial oven temperature of 100 °C held for 1 min, with a temperature ramp of 12 °C min to 300 °C and held for 5 min. Helium was used as a carrier gas at a flow rate of 1.5 mL/min, and sample separation was achieved on a 30 m Rxi-5MS (0.25 mm internal diameter, 0.25 um thickness) fused silica column (Restek, Bellafonte, Pennsylvania).

### 2.5. Plasma Glucose and Insulin

Plasma glucose concentrations were measured using the YSI 2300 STAT Plus Glucose and Lactate analyzer YellowSprings, OH, USA (YSI 2300). Plasma concentrations of insulin were determined by enzyme linked immunosorbent (ELISA) assay with the ultrasensitive kit from Mercodia (Winston Salem, NC, USA). HOMA-IR was calculated according to the formula: fasting insulin (microU/L) × fasting glucose (nmol/L)/22.5 [17].

### 2.6. Statistical Analysis

Data are expressed as mean ± standard error of the mean (s.e.m), while normality and distribution were examined using the Shapiro–Wilk test. In addition, analysis of covariance using baseline values for each outcome as a covariate and mixed measures analysis of variance (time) with one between-subject factor (group) were used to compare the changes in both the BCAA-restricted diet and control diet. The significance level was defined as *p* < 0.05, and all the statistical analyses were preformed using Graph Pad Prism 8.01 (La Jolla, CA, USA).

## 3. Results

Participants observed an above 95% consumption rate in both the BCAA-restriction diet and the control diet for all meals and supplements. Twelve healthy individuals (8 male/4 female); mean age of 26 y, physiological characteristics are shown in (Table 2). As expected, both the BCAA-restriction diet and control diet had no effect on weight or body–mass index (BMI) over seven days (Table 2).

### 3.1. BCAA Levels

**Figure 1:**
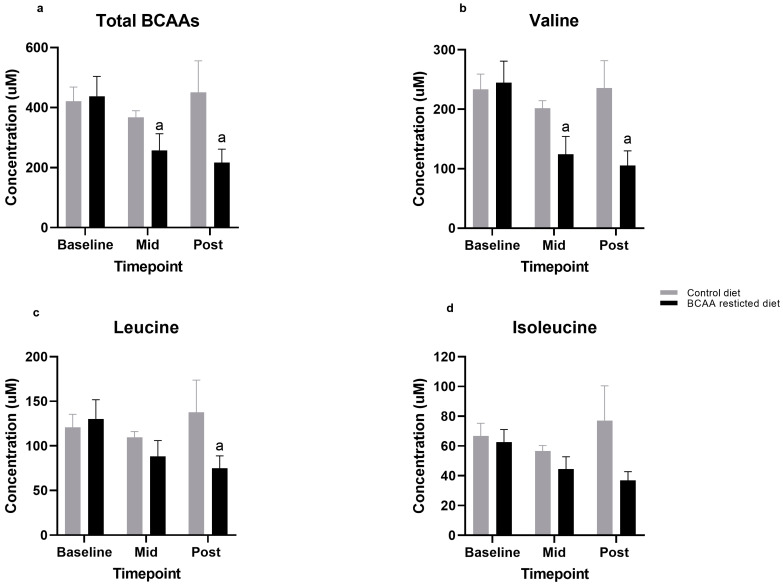
(**a**–**d**). Comparison of Total and individual Branched-chain amino acid (BCAA) levels between control and BCAA-restricted diets. Values are means (standard error of mean). ^a^ significantly different from baseline, *p* < 0.05. Mid: Day 3, Post: Day 7.

After just three days, the BCAA-restricted diet elicited a significant 41% decrease in total circulating BCAA concentrations (437 ± 60 to 257 ± 51 µmol/L; *p* = 0.02). Furthermore, by day seven, total circulating BCAA concentrations had significantly decreased by 50% from baseline (437 ± 60 to 217 ± 40 µmol/L; *p* = 0.02). In contrast, the control diet showed a negligible, and non-significant increase of 7% in total circulating BCAA concentrations (421 ± 43 to 451 ± 96 µmol/L; *p* = 0.97) (Figure 1a).

### 3.2. Individual Amino Acids

Of the three BCAAs, valine exhibited the greatest decrease over the seven days, by ~57% from baseline during the restricted diet: (245 ± 33 to 105 ± 23 µmol/L; *p* < 0.0001) (Figure 1b). This was followed by leucine, with a decrease of 42% from baseline (130 ± 20 to 75 ± 13 µmol/L; *p* < 0.04) (Figure 1c). Although the levels of isoleucine were also lowered by 41% this was not statistically significant: (63 ± 8 to 37 ± 5 µmol/L; (*p* = 0.96) (Figure 1d). In contrast, the control diet yielded no differences in any BCAA (Figure 1b–d).

### 3.3. Other Amino Acids

**Figure 2:**
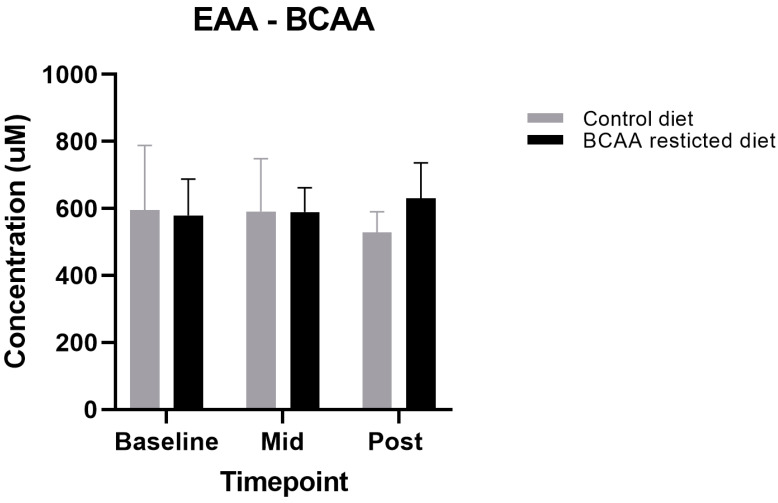
Comparison of Essential amino acids (EAA)-Branched-chain amino acid (BCAA) levels between control and BCAA-restricted diets. Values are means (standard error of mean). *p* < 0.05. Mid: Day 3, Post: Day 7.

**Figure 3:**
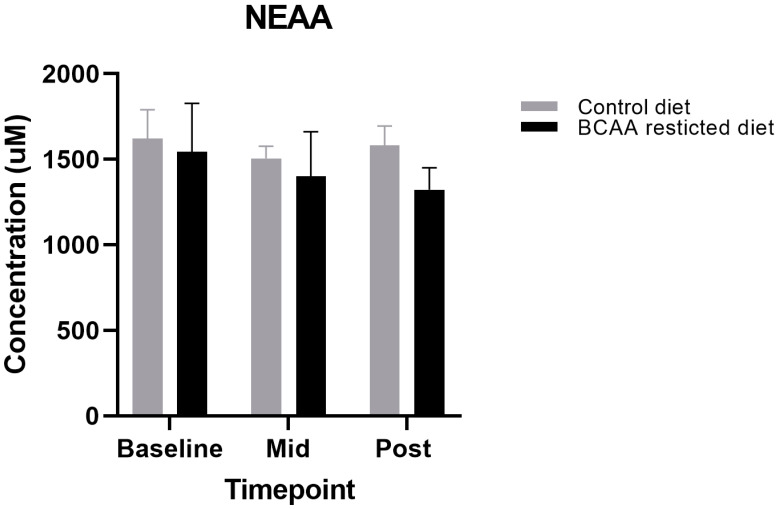
Comparison of Non-essential amino acid (NEAA) levels between control and Branched-chain amino acid (BCAA)-restricted diets. Values are means (standard error of mean). *p* < 0.05. Mid: Day 3, Post: Day 7.

Although the primary aim of this study was to lower circulating BCAA concentrations, we also aimed to design a diet that met protein requirements and was iso-nitrogenous between the restricted diet and control, due to the crucial role of BCAA in regulating muscle metabolism. This included the non-essential amino acids (NEAA) and essential amino acids minus the three BCAAs (EAA—BCAAs). We found no significant differences between all the NEAA and EAA—BCAA during both the BCAA-restricted and control diets (*p* > 0.05) (Figure 2 and Figure 3).

### 3.4. Glucose, Insulin, and HOMA-IR Levels

During the BCAA-restricted diet, individuals demonstrated a tendency towards decreasing fasting glucose over seven days; (mean difference = 0.47 ± 0.2 mmol/L; *p* = 0.07). However, the control diet produced a less evident decrease (mean difference = 0.25 ± 0.2 mmol/L; *p* = 0.42) (Table 3). Intriguingly, the BCAA-restriction diet also tended to reduce HOMA-IR values (1.5 ± 0.2 to 1.0 ± 0.1; *p* = 0.096). Conversely, during the control diet, HOMA-IR increased over seven days compared with baseline: (1.0 ± 0.2 to 1.2 ± 0.2; *p* = 0.24) (Table 3).

## 4. Discussion

Elevated circulating BCAA concentrations are associated with T2D, obesity and IR [18] but to establish causal link, an intervention study involving dietary restriction of BCAA, specifically, is needed in humans. The aim of this study therefore was to investigate the feasibility and safety of specifically reducing circulating BCAA concentrations in healthy individuals using a novel dietary intervention that achieved energy balance, provided adequate protein, while maintaining iso-nitrogenous and iso-caloric levels between the intervention and control arms.

Although BCAA-restricted diet studies have been successfully undertaken in animal models to investigate the pathogenic mechanisms of BCAA in regulating glucose homeostasis and weight [8,19], allied dietary intervention studies in human subjects have been lacking [9,20]. In designing our BCAA-restricted diet, we considered the potential impact BCAA restriction would have upon skeletal muscle mass, which represents the largest storage pool of amino acids in the body [21]. Indeed, EAAs including BCAAs are crucial in maintaining muscle mass [22]. In addition, MSUD has been associated with muscle atrophy and dysfunction due to BCAA restriction [23]. Thus, it was imperative that our BCAA-restricted diet be designed with two key elements. First, to restrict BCAA levels by 75% while maintaining overall protein intake, we carefully selected foods with a low BCAA to total protein ratio (i.e., fruit and vegetables) and acquired commercially available amino acid mixtures free of BCAAs. Secondly, we used naturally low protein foods, and commercially available low/no protein foods (e.g., bread and pasta) to maintain energy balance via fat and carbohydrate intake. The combination of these two factors would prevent the volunteers entering a catabolic state. Our study succeeded in manipulating circulating BCAA levels through specifically targeting dietary BCAA intake. The findings of this study are consistent with others, several studies have reported increased circulating BCAA levels in individuals with higher dietary BCAA intake when compared with individuals with lower dietary BCAA consumption [2,3,24].

Interestingly, individually the BCAA levels varied in their significance. Valine represented the most significant decrease of all three BCAAs. Our findings are consistent with previous studies, Lu et al. reported concentrations of valine led to a significant improvement in risk prediction of incidence diabetes when compared to isoleucine and leucine [25]. Furthermore, studies have demonstrated higher levels of valine when compared to isoleucine and leucine in type 2 diabetes patients, and even suggested valine alone as a novel biomarker of type 2 diabetes [26]. As of yet, there are no studies looking at the effects of manipulating circulating levels of valine alone. Future studies are needed to determine the individual effects of systematic BCAA restriction.

Clinical and pre-clinical studies have consistently associated elevated circulating BCAAs as a cause of IR [27]. Several mechanisms have been suggested including, dysregulation of cellular pathways associated with hepatic and muscle glucose disposal via BCAAs negative feedback actions through stimulation of the mechanistic target of rapamycin (mTOR), and inhibitory crosstalk to proximal insulin signaling pathways i.e., the insulin receptor (IR) [4,28]. Thus, we hypothesized that a reduction in circulating BCAAs would also improve insulin sensitivity. Although it was not the main aim of this study (i.e., underpowered to robustly detect effects on HOMA-IR), we nevertheless observed that BCAA restriction was associated with a decrease in HOMA-IR levels in healthy subjects assigned to the BCAA-restricted diet over seven days, with a *p*-value approaching significance. The findings of our study are consistent with previous BCAA-restriction studies in animals which reported improved insulin sensitivity in mice [29].

The BCAA-restricted diet reduced circulating BCAA levels by 50% while maintaining iso-nitrogenous, iso-caloric dietary intakes, and while meeting the RDA for protein requirements, our novel dietary intervention was specifically targeted to BCAA restriction. Intriguingly, that serum insulin and HOMA-IR tended improve with no concurrent weight change, points towards efficacy—even in non-obese younger individuals. Although larger early phase clinical trials are required, the present pilot study represents a novel dietary means by which to reduce BCAA, and as such, provides a blueprint for a potential dietary intervention in obesity/diabetes.

Several limitations are noted, since this was a pilot study, the number of participants recruited was relatively small and as such underpowered to robustly investigate the effects upon HOMA-IR. Furthermore, due to unforeseen circumstances we were unable to use the same participants for both the BCAA-restricted and control diets. It should also be noted that low BCAA food products as well as the low BCAA supplement drink Lophlex, are very costly, this will have implications on larger studies. In addition, our study was a short-term intervention, and adherence to this diet may change over longer durations. That said, the diet was straightforward to prepare while also still being palatable since we observed over a 95% compliance rate. Moreover, our dietary model successfully, and specifically, lowered circulating BCAA concentrations by 50% over 7 days while also maintaining energy intake and meeting protein requirements. Furthermore, the BCAA-restricted diet was associated with a marginal (albeit non-significant) improvement in HOMA-IR, in the absence of weight loss, adverse effects, or safety concerns. Therefore, despite this being a short-term dietary manipulation study, the present paradigm provides a framework forming the basis for future interventional studies to ascertain causal links between BCAA levels and IR; perhaps to improve and treat IR and/or type 2 diabetes.

## Figures and Tables

**Table 1:** Menu and timetable of participant’s meals between BCAA-restricted diet and control diet.

Diet	Control Diet		BCAA-Restricted Diet	
Menu	Food	Supplement	Food	Supplement
Breakfast	Loprofin sliced loaf with peanut butter.	34% of daily amount to be consumed. Each sachet contains Asn, Glu and Gly.	Loprofin sliced loaf with peanut butter.	34% of daily amount to be consumed. Each sachet contains Asn, Glu, Gly, Iso, Leu and Val.
Snack 1	Apple and cinnamon muffin made with Loprofin mix.	No supplement taken.	Apple and cinnamon muffin made with Loprofin mix.	No supplement taken.
Lunch	Carrot and coriander soup made with Snowpro with a cheese sandwich.	21% of daily amount to be consumed. Each sachet contains Asn, Glu and Gly.	Carrot and coriander soup made with Snowpro with a cheese sandwich.	21% of daily amount to be consumed. Each sachet contains Asn, Glu, Gly, Iso, Leu and Val.
Snack 2	Apple and cinnamon muffin made with Loprofin mix.	No supplement taken.	Apple and cinnamon muffin made with Loprofin mix.	No supplement taken.
Dinner	Vegetable curry with either Loprofin rice (or pasta).	26% of daily amount to be consumed. Each sachet contains Asn, Glu and Gly.	Vegetable curry with either Loprofin rice (or pasta).	26% of daily amount to be consumed. Each sachet contains Asn, Glu, Gly, Iso, Leu and Val.
Snack 3	Mixed chocolate and almond snack.	19% of daily amount to be consumed. Each sachet contains Asn, Glu and Gly.	Mixed chocolate and almond snack.	19% of daily amount to be consumed. Each sachet contains Asn, Glu, Gly, Iso, Leu and Val.

BCAA, branched-chain amino acid, Asn, asparagine, Glu, glutamine, Gly, glycine, Iso, isoleucine, Leu, leucine, Val, Valine.

**Table 2:** Participants characteristics.

	Control		BCAA-Restricted	
	Baseline	Day 7	Baseline	Day 7
Age (Years)	27 ± 7	*	24 ± 3	*
Height (m)	161.4 ± 12	*	173.4 ± 7	*
Weight (kg)	62.2 ± 5	62.2 ± 5	70.2 ± 9	70.2 ± 9
BMI (Kg·m^−2^)	22.8 ± 6	22.8 ± 6	22.2 ± 5	22.2 ± 5
BP	120/82	118/69	116/83	110/79

BCAA, branched-chain amino acid. * Same as baseline, values are means (standard error of mean).

**Table 3:** Comparison of Glucose and HOMA-IR levels between Branched-chain amino acid (BCAA)-restricted and control diets. HOMA-IR, Homeostatic Model Assessment of Insulin Resistance.

	Control			BCAA-Restricted		
	Baseline	Day 7	*p* Value	Baseline	Day 7	*p* Value
Glucose (mmol/L)	5.5 ± 0.2	5.2 ± 0.3	0.41	5.3 ± 0.2	4.9 ± 0.3	0.07
Insulin (miliU/L)	3.8 ± 0.5	5.5 ± 0.9	0.26	6.3 ± 1.0	4.9 ± 0.6	0.17
HOMA-IR	1.0 ± 0.2	1.3 ± 0.1	0.24	1.5 ± 0.2	1.0 ± 0.1	0.09

## Data Availability

The data presented in this study are available on request from the corresponding author.

## Abbreviations

T2D	Type 2 diabetes
IR	Insulin resistance
BCAA	Branch chain amino acid
HOMA-IR	Homeostatic Model Assessment of Insulin Resistance
EAA	Essential amino acids
NEAA	Non-essential amino acids
BMI	Body mass index
RMR	Resting metabolic rate
PAL	Physical activity levels
IPAQ	International Physical Activity Questionnaire
BP	Blood pressure
